# Granulocytes in Helminth Infection - Who is Calling the Shots?

**DOI:** 10.2174/092986712799828337

**Published:** 2012-04

**Authors:** BL Makepeace, C Martin, JD Turner, S Specht

**Affiliations:** 1 Department of Infection Biology, Institute of Infection & Global Health, University of Liverpool, Liverpool L69 7ZJ, UK; 2 USM 7245 MCAM, CNRS MNHN, Muséum National d’Histoire Naturelle, 61 rue Buffon, 75231 Paris Cedex 05, France; 3 Department of Molecular and Biochemical Parasitology, Liverpool School of Tropical Medicine, Liverpool, L3 5QA, UK; 4 Institute for Medical Microbiology, Immunology and Parasitology, University Hospital Bonn, 53105 Bonn, Germany

**Keywords:** Helminths Eosinophil, neutrophil, basophil, filariasis, T_H_2 response, reactive oxygen species, reactive nitrogen species, nitric oxide.

## Abstract

Helminths are parasitic organisms that can be broadly described as “worms” due to their elongated body plan, but which otherwise differ in shape, development, migratory routes and the predilection site of the adults and larvae. They are divided into three major groups: trematodes (flukes), which are leaf-shaped, hermaphroditic (except for blood flukes) flatworms with oral and ventral suckers; cestodes (tapeworms), which are segmented, hermaphroditic flatworms that inhabit the intestinal lumen; and nematodes (roundworms), which are dioecious, cylindrical parasites that inhabit intestinal and peripheral tissue sites. Helminths exhibit a sublime co-evolution with the host´s immune system that has enabled them to successfully colonize almost all multicellular species present in every geographical environment, including over two billion humans. In the face of this challenge, the host immune system has evolved to strike a delicate balance between attempts to neutralize the infectious assault versus limitation of damage to host tissues. Among the most important cell types during helminthic invasion are granulocytes: eosinophils, neutrophils and basophils. Depending on the specific context, these leukocytes may have pivotal roles in host protection, immunopathology, or facilitation of helminth establishment. This review provides an overview of the function of granulocytes in helminthic infections.

## INTRODUCTION

Granulocytes are a class of leukocytes characterized by the presence of lobulated nuclei and secretory granules in their cytoplasm. They are generated from hematopoetic stem cells, which can differentiate into common lymphoid progenitor cells or common myeloid progenitor cells. Hematopoetic stem cells, once developed into progenitors, acquire specialized features of a particular cell type while losing lineage differentiation potential [1]. While lymphoid lineage cells include T, B and natural killer cells, differentiation of the myeloid lineage gives rise to mast cells, megakaryocytes, erythrocytes, dendritic cells (DC), macrophages and granulocytes [2]. The use of chemical dyes for selective cell staining enabled Paul Ehrlich in the late 19^th^ century to describe three different types of granulocytes (eosinophilic, neutrophilic and basophilic polymorphonuclear cells) [3]. They are highly conserved, with relatively simple forms found in some invertebrates (such as molluscs), while all vertebrates are endowed with at least one type of granulocyte. Under normal conditions the human blood contains up to 50% neutrophils; whereas eosinophils comprise only 1-5%, and basophils less than 1% of circulating blood leukocytes. 

Granulocytes can secrete a wide variety of mediators without *de novo* protein synthesis; a particularly important characteristic that highlights their key role in innate and adaptive immune functions. The capability of granulocytes, most notably eosinophils, to release toxic cationic proteins has been considered historically as an effector mechanism against extracellular organisms [4], although these molecules have also been implicated in tissue damage. Thus, granulocyte-mediated immunopathology is observed in hyperre-activity during some nematode infections and is also frequently manifested in allergic responses such as asthma [5]. The release of granule proteins can be induced through binding of antigen-IgE complexes to the high affinity IgE receptor (FcεRI) that triggers a tightly controlled phosphorylation cascade [6]. The classical view of granulocyte function has been reconsidered over the last decades, as new data have demonstrated that this cell type has roles other than that of a terminal effector cell [5, 7]. 

The functional analysis of granulocytes in helminth infections relies on interventional studies and includes suitable animal models in conjunction with immunological or genetic tools to interfere with normal granulocyte development and function. Despite the caveat that laboratory model organisms may not always be the natural host of the parasite, and therefore cannot represent all processes observed in natural infections of livestock or humans, clearly many paradigms translate well between the species and have led not only to greater understanding of parasitic diseases, but in several cases, to successful therapies. This review is not intended to cover the entire field of granulocyte biology but to focus on their functions in relation to a particularly complex foe, the helminth parasites. In particular the role of eosinophils, basophils and neutrophils in host protection, immunopathology or facilitation of helminth establishment will be discussed.

## T_H_2 IMMUNITY TO HELMINTH INFECTIONS

**Fig. (1). T cell mediated effector mechanisms against pathogens.**
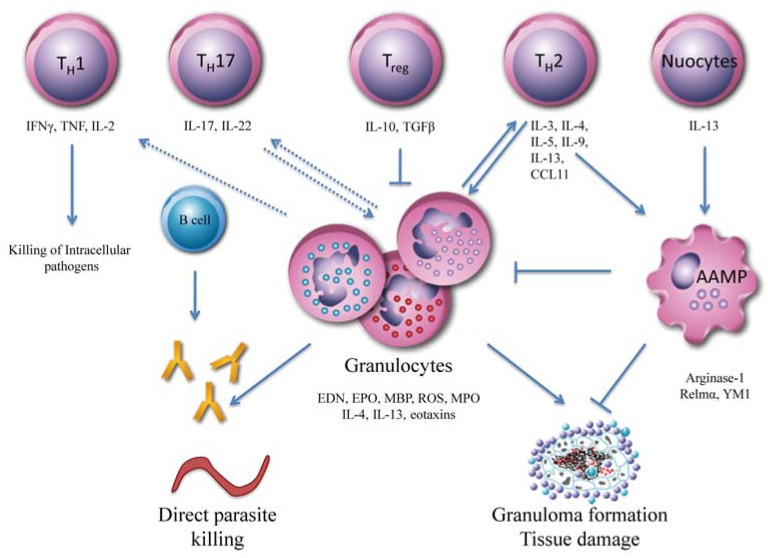
Innate immune mechanisms are the first to respond to place against infection. They consist of soluble factors, such as complement proteins, together with many cellular components including mast cells, macrophages, dendritic cells, natural killer cells and granulocytes (eosinophils, neutrophils and basophils). Adaptive immune responses develop more slowly, but result in increased antigen specificity and immunological memory, and are orchestrated by CD8^+^ T cells, CD4^+^ T cells and B lymphocytes. Among the T-helper (T_H_) cells, T_H_1 cells are protective against intracellular pathogens and T_H_17 cells against extracellular bacteria, whereas T_H_2 cells play a central role against extracellular pathogens, such as large helminths. The induction and maintainance of a T_H_2- type environment is strongly supported by recently discovered nuocytes and T_H_2 responses also play an important role in wound healing by the induction of alternatively activated macrophages (AAMP). Excessive effector T cell responses are controlled by regulatory T cells (Treg) in order to prevent immunopathology, as seen in allergic reactions (overinduction of T_H_2) or autoimmune diseases (overinduction of T_H_1 or T_H_17).

In response to an infection, a variety of cells becomes activated and collaborates in the effort to control and eliminate invading pathogens (see Fig. **1**). T_H_1 cells mainly produce IFNγ, which is important for classical macrophage activation and the clearance of many intracellular microbes. Large extracellular pathogens face immune mechanisms that are of a T_H_2-type, characterized by an elevation of peripheral blood eosinophilia and accompanied by profound increases in cytokine production Interleukin (IL)-3, IL-4, IL-5, IL-9, IL-13) and granulocyte macrophage colony-stimulating factor (GM-CSF) as well as induction of the antibody isotypes immunoglobulin (Ig) G1, IgG4 and IgE. In mice lacking the key component of T_H_2-type immunity, *i.e.* CD4^+^ T cells, protective immunity to the nematodes *Nippostrongylus brasiliensis *[8], *Litomosoides sigmodontis* [9] and other helminths [10] is lost, providing evidence to support the importance of such responses in parasite clearance. Historically, it was hypothesized that T_H_2 responses are induced by suboptimal antigen presentation and consequently, ineffective stimulation of the T_H_1 pathway. However, helminth products can drive T_H_2 immunity even in the presence of T_H_1 inducers. For example, when stimulated with *Schistosoma mansoni* soluble egg antigen (SEA), DC are able to induce T_H_2 responses in the presence of bacterial T_H_1 stimuli [11]. 

A more complex picture is now emerging, in which IL-25, IL-33 and thymic stromal lymphopoietin (TSLP) [12] have been shown to be strong inducers of T_H_2-type immunity. Blockade of IL-25 in mice, for example, results in reduced hyper-reactivity and inflammation in a model of ovalbumin-induced airway hypersensitivity [13]. This was associated with reduced eosinophil influx into the lung and suppression of IgE production. The effects induced by IL-25 seem to be very similar to those induced by IL-33, in that administration of both cytokines causes eosinophilia, splenomegaly, IgE secretion, production of other T_H_2-type cytokines, goblet hyperplasia and mucus production [14, 15]. TSLP is yet another potent T_H_2-type inducer found particularly in epithelial cells of the lung, skin and gut [16]. It belongs to the IL-7 family, and among other functions sensitizes DC to promote a T_H_2 developmental pathway. In depth investigation of these molecules led to the identification of lineage-marker negative cells; i.e., nuocytes, natural helper cells, innate helper cells and multi-potent progenitor cells, which are intimately involved in the generation of T_H_2-type responses *via* the production of IL-13. The role of these new cell types in immune responses has been extensively discussed in [17]. As this review will focus on only those aspects that are relevant to granulocyte function, one should refer to [10] for a thorough overview of more general features of T_H_2 immunity.

The host-parasite interface, which is predominantly in the gut or peripheral tissues, is the location where downstream effector mechanisms of T_H_2-type responses are developed for parasite expulsion or killing. In the gut, IL-13 promotes goblet cell hyperplasia and augments mucus production [18], contributing to worm expulsion. For the targeting of parasites in peripheral tissues, antibody production and recruitment of effector cells that release cytotoxic molecules upon activation (*e.g*., *via* cross-linking of Fc-receptors) eventually damage the parasite, although if these processes are not properly controlled, they can also harm the host.

In addition to the well-described effector function against helminthic parasites, T_H_2 responses are part of adaptive and therefore memory responses, which also result in tissue repair mechanisms. Since tissue destruction is a common manifestation of many helminth infections, limiting parasite-mediated damage is critically important in mitigating disease sequelae. For instance, both *Ascaris* and *Nippostrongylus* can lead to damage of the lung tissue during migration through the host. The T_H_2 cytokines, IL-4 and IL-13 for example, are potent inducers of molecules involved in wound healing processes, such as resistin-like-molecule-α (RELMα ), arginase, matrix metallopeptidase 12 (MMP12) and triggering receptor expressed on myeloid cells 2 (TREM-2) [19]. Along with such T_H_2 responses comes the alternative activation of macrophages which has been shown for many helminth infections. Indeed, although evolutionary hypotheses can be difficult to test, one intriguing proposal is that T_H_2 responses may have emerged as a tissue repair mechanism rather than being primarily anti-parasitic [20]. 

## EOSINOPHILS

Eosinophils are mainly associated with allergic responses (such as asthma), viral infections or helminthiases. They are equipped with a variety of receptors (including those for chemokines, cytokines, immunoglobulins, complement and serine proteases) that enable them to be recruited into affected tissue sites and release granule contents [5, 21]. Mainly IL-5, but also IL-3 and GM-CSF promote eosinophil development whereas recruitment is mediated (among others) by chemokine ligand 11 (CCL11) and CCL26 (eotaxins), which in turn are controlled by IL-13. Most notably in helminth infections, ligation of parasite-specific immunoglobulins to Fc receptors is critically important for a process known as antibody-dependent cytotoxicity (ADCC), which results in activation of the eosinophils. However, they can also be activated by a number of other mediators, such as Toll-like receptor (TLR) ligands, chemokines and cytokines. Activation leads to classical exocytosis, in which the complete granule contents are released by fusion with the cellular membrane; and cytolysis, in which the plasma membrane ruptures and granules are deposited extracellularly (extensively reviewed in [5]). A highly regulated process termed “piecemeal degranulation” can occur also in eosinophils. This is characterized by emergence of vesicles from the granules, which traffic through the cytoplasm to the cell membrane for release of transported contents [22]. Although these different pathways to degranulation are likely to be tightly controlled, the exact mechanisms remain unclear.

Eosinophilic granules are primarily composed of the following cytotoxic, cationic proteins: major basic protein (MBP), eosinophil peroxidase (EPO), eosinophilic cationic protein (ECP) and eosinophil-derived neurotoxin (EDN) [5]. They are also equipped with a number of cytokines, chemokines and growth factors for very rapid release, which can alert the immune system to combat pathogenic organisms. Importantly, several of these mediators have additional functions, such as communicating with other cells by signaling interactions, including endothelial cells and mast cells (for more detailed review see [21]). Although the amounts produced are not comparable to the levels released by T cells, the fact that such molecules exist in preformed granules allows eosinophils to act as excellent gatekeepers during parasite entry to rapidly initiate immune responses. 

Interventional studies on eosinophils have used a variety of approaches: eosinophil or IL-5 depleting antibodies; genetic modification leading to overexpression of IL-5 (IL-5 transgenic hypereosinophilic mice) or *vice-versa* IL-5 deficiency; abrogation of the eosinophil lineage (ΔdblGATA), or suicide activation during development induced by diphtheria toxin (TgPHIL) [23]. In addition, deficiency of recruitment factors such as the chemokine CCL11 [24] leads to reduced numbers of eosinophils. Information on the functional relevance of specific eosinophil-related mechanisms can be obtained from granule protein deficiency (*e.g.* MBP [25], EPO [26]) or by the use of µMT mice [27], which do not produce functional antibodies and thus cannot mediate ADCC. 

## NEUTROPHILS

Neutrophils are the most numerous type of leukocytes, and with a circulating half-life of only 6–8 hours their lifespan is shorter compared to that of eosinophils. The differentiation factors for neutrophils are G-CSF, IL-6, GM-CSF and IL-3 while multiple inflammatory mediators, including leukotriene B4, complement component C5, IL-8 and TNFα are able to induce neutrophilia [28]. When neutrophils are recruited to an inflamed compartment, apoptosis can be inhibited resulting in extended survival. Neutrophils are classically characterized as phagocytic cells that remove and destroy invading microorganisms; hence neutrophils are a vital component of the innate immune system and play a crucial role in host defence [29]. This is illustrated by the potentially fatal complications that arise in neutropenic individuals, such as patients with WHIM syndrome [30]; or by the morbidity associated with clinical conditions in which genetic polymorphisms give rise to abnormal neutrophil physiology, for instance, as leukocyte adhesion deficiency 1 (LAD1) and chronic granulomatous disease [31].

Neutrophils are the first cell type recruited to the site of an acute inflammatory response and are characterized by their ability to act as phagocytic cells, to release lytic enzymes, and to produce reactive oxygen species with anti-microbial potential [32, 33]. These released products may act in conjunction with cells resident in the affected tissue, such as macrophages and mast cells, to amplify the initial inflammatory response [34] and induce the recruitment of additional neutrophils, other leukocyte populations (including monocytes and lymphocytes), and (in T_H_2 scenarios) eosinophils and basophils. It has become apparent that neutrophils are important mediators of the T_H_17 pathway of resistance to pathogens [35]. This arm of the immune system is characterized by a sustained inflammatory milieu that also may give rise to an adaptive immune response. However, in certain circumstances, the excessive activation of such cellular defences may cause inappropriate damage to host tissues. This has been linked to the pathogenesis of a variety of diseases, including adult respiratory distress syndrome [36], chronic obstructive pulmonary disease [37], and sudden-onset fatal asthma [38]. Because of their predominant role in phagocytosis of microbial pathogens, neutrophils have often been overlooked in helminth infections, although this is beginning to be addressed in animal models and hence indications for their involvement in immune responses to helminths will be discussed. Tools for research on neutrophils comprise mainly antibodies against GR-1, Ly6G, PMN or G-CSF; whereas in genetic mouse models, CXCR2- and NE-deficient [39] mice have been used. 

## BASOPHILS

Since they are comparatively rare, the immunology of basophils has been neglected for many years, but these cells are now recognized for their role in initiating immune responses in addition to their effector function. They share many features with mast cells, including FcεRI expression and the capacity to secrete reactive oxygen and nitrogen species [40], T_H_2 cytokines, and histamine. However, in contrast to mast cells, which are located in tissues and have the potential to proliferate in response to IL-3, IL-4 and IL-9, basophils circulate as fully matured cells in the blood. IL-3 is the main factor responsible for their optimal activation, population expansion and survival [41], although more recently it has been shown that TSLP acts in concert with IL-3, to propagate these effects [42]. Interestingly, in response to TSLP but independently of IL-3 a different phenotype of basophils was induced, which exhibited reduced degranulation capacity but higher IL-4 and IL-6 production; moreover the gene expression profile in these cells was enriched in cell communication and adhesion.

Basophils are characterized by the presence of basophilic granules and surface expression of high affinity Fcα RI for binding of IgE in addition to cytokine receptors, chemokine receptors and complement receptors [43]. Upon ligation they release chemical mediators, such as leukotriene C4 and histamine, and particularly the T_H_2 cytokines IL-4 and IL-13, which implicates basophils in immune responses elicited by helminthiases [44]. Basophils display a remarkable potential to contribute to the symptoms of allergic inflammation through the release of histamines and leukotriene, and have been proposed to play a key role by inducing class switching to IgE in B cells [45]. Recent studies suggest that basophils induce IgE-mediated chronic allergic inflammation [46] and IgG_1_-mediated systemic anaphylactic shock [47]. Depletion of basophils during IgE-mediated dermatitis reduced the number of eosinophils and neutrophils in the skin, suggesting an important role for basophils in the initiation of allergic responses and more importantly, in interactions between granulocytes [48]. Several studies have indicated that basophils may be important in the T_H_2 polarisation of the immune response [49], mainly by secreting IL-4. This will be discussed in the following section. 

Tools for the study of basophils mainly consist of antibody-mediated depletion targeting CD200 receptor 3 (Ba103) or the high affinity Fcα receptor (MAR-1) [49]. Recently, an inducible basophil-deficient mouse was generated (*Mcpt8*^DTR^), in which injection of diphtheria toxin led to a transient depletion of basophils [50]. In addition, mice that have the Cre recombinase expressed under the control of the carboxypeptidase A3 promotor have been crossed to mice bearing a floxed allele of Mcl-, also showed a marked deficiency of basophils [51]. Reporter mice, in which either IL-4 [52] or basophil-specific protease expression [53] can be monitored, offer the possibility to track activated basophils *in vivo*.

## RECENTLY RECOGNIZED FUNCTIONS OF GRANULOCYTES

In addition to the classical downstream effector functions of granulocytes, such as ADCC, which direct the release of toxic molecules against pathogens, granulocytes express a particularly rich repertoire of TLRs, the key molecules that mediate innate immune responses. These receptors recognize ligands from infectious agents termed “pathogen-associated molecular patterns” (PAMPs). Binding of PAMPs to TLRs activates cytoplasmic adaptor molecules that trigger a protein kinase cascade, leading to induction or suppression of inflammatory response genes. From the thirteen TLRs identified to date, most are expressed by granulocytes (see Table **1**). The majority of characterized TLR ligands are derived from viruses or unicellular pathogens, although more recently, a small number of PAMPs from helminths has been discovered. These include molecules from the trematode *Schistosoma mansoni*: the lipid lysophosphatidylcholine (lyso-PC) [54] and the glycan lacto-*N*-fucopentaose III [55], which are ligands for TLR-2 and -4, respectively. While lyso-PC is involved in eosinophil recruitment, the lysophosphatidylserine (lyso-PS) [56], another ligand for TLR-2, is able to skew immune responses towards IL-10 production and T cell hyporesponsivness. Such suppression is an important mechanism during apoptosis by the host´s own phosphatidylserine [57]. This shows that different molecules can stimulate the same TLRs with varying outcomes, whereas a single molecule, irrespective of whether the molecule is derived from the parasite or the host, can induce a similar type of response, *i.e.* immunosuppression. Co-evolution of the host and parasite has honed many of such immune evasion strategies in order for the parasite to thrive in its host. In nematodes, the excretory-secretory glycoprotein ES-62 from *Acanthocheilonema viteae *can also bind to TLR-4 [58], while the polysaccharide chitin, which is widespread among nematodes and other non-vertebrate phyla (Table **1**), is a ligand for TLR-2. In addition, the unique biology of certain filarial nematodes (*e.g.*, *Wuchereria bancrofti* and *Onchocerca volvulus*), which have an obligate relationship with a bacterial symbiont (*Wolbachia*; see last section), results in the activation of TLRs that recognise bacterial PAMPs during infections with these species [59, 60]. Whereas ligation of ES-62 to TLR-4 leads to deactivation of mast cells and therefore prevents immune-mediated damage, a converse effect has been attributed to *Wolbachia* recognition by TLRs in filarial infections, which appears to be a strong inducer of pathology in humans [60]. Importantly, despite the apparent ability of TLRs to recognize helminth products, there is little evidence that this process translates into induction of the adaptive T_H_2 pathway. Indeed, other molecules, such as C-type lectin receptors may be more important than TLRs in this context, but the precise mechanisms remain elusive [10]. For a more comprehensive overview of the innate recognition of helminths by the mammalian immune system, the reader is directed to other recent reviews [61, 62]. 

Irrespective of our ignorance of the exact pathways involved, several line of evidence suggest that granulocytes are indeed key participants in bridging innate and adaptive immunity. It is now widely accepted that eosinophils [63], neutrophils [64] and basophils [65] not only have the ability to sense and react to PAMPs but also to phagocytose, process and present antigen to naïve T cells. Whereas presentation of helminth-derived antigens by neutrophils remains to be demonstrated, eosinophils from healthy human donors stimulated with *Brugia malayi* extracts or isolated from rodents after infection with *B. malayi*, increased their surface MHC class II expression and upregulated costimulatory molecules such as CD40 and CD86 [66, 67]. Furthermore, eosinophils exposed to *Strongyloides stercoralis* were capable of inducing IL-5 production by T cells *in vitro* [68] and *in vivo* after adoptive transfer of antigen-exposed eosinophils [69]. They may also play a role in maintaining T_H_2 responses by secretion of IL-4 [70].

Recently, a major focus has been on basophils, which have been reported to act as antigen-presenting cells and to be an important source of IL-4 and IL-13, prerequisites for the induction of T_H_2 responses. For example, depletion of basophils results in impaired protection against several parasites; *e.g.* the gastrointestinal helminths *N. brasiliensis* [71] and *Trichuris muris *[19]. In the latter study, it was also shown that basophils expressed MHC class II and IL-4 and were recruited to the lymph nodes upon challenge of mice with *S. mansoni* eggs, thereby having the potential to interact with naïve T cells in peripheral lymphoid tissues. A major secreted product of *S. mansoni *eggs, alpha-1, also known as IL-4-inducing principle of schistosome eggs (IPSE), has been demonstrated to activate mouse [72] and human basophils, causing release of IL-4 *in vitro *[73, 74], which was dependent on IgE crosslinking [74]. Despite this induction of an immune response, a recent study by Sullivan and colleagues [53] reported that in accordance with [75], basophils may not be implicated in protective responses during primary helminth infection. A similar observation was made in murine infection with the filarial parasite *L. sigmodontis* [76], since removal of basophils did not alter the outcome of infection, despite a reduction in T_H_2 immunity. However, basophils may play a major role in type-2-mediated secondary infection in conjunction with CD4^+^ T cells, as depletion of IL-4 and IL-13 in both basophils and CD4^+^ T cells was necessary to abrogate protection. It was elegantly shown that IL-4 production in basophils and also eosinophils was restricted to affected tissues; *e.g.,* the lung in primary *N. brasiliensis* infection [53]. However, other studies have shown that T_H_2 induction can occur in the absence of basophils. IL-3 deficient mice, for example, show an impaired recruitment of basophils to lymph nodes, but this did not affect elicitation of T_H_2 responses upon infection with *N. brasiliensis* [77]. Indeed, it has been suggested that IL-4 produced by basophils may be more important in maintaining T_H_2 immunity, rather than inducing this response [7]. Hence, the *in vivo* relevance of granulocytes as antigen-presenting cells or as T_H_2 inducers during helminth infections requires further investigation.

## ROLE OF GRANULOCYTES AGAINST TREMATODES, CESTODES AND NEMATODES

### Trematodes

Commonly known as “flukes”, trematodes have a dorsoventrally flattened body with bilateral symmetry, ranging from a few millimeters up to 7-8 cm in size. In general, snails are involved as intermediate hosts and the development includes several larval stages before reaching adulthood. Trematodes of medical importance include *Schistosoma* spp. (blood flukes), *Clonorchis* *sinensis*, *Fasciola* spp. (liver flukes) and *Paragonymus* *westermani *(lung fluke), of which *Schistosoma* spp. are the best-studied parasites. Five species infect humans, with *S. mansoni, S. japonicum, S. intercalatum* and* S. mekongi* inducing intestinal and liver disease, whereas *S. haematobium* affects the urinary tract. Following the release of parasite eggs into water, a free-living motile larval stage (miracidium) hatches to infect freshwater snails. After transformation and asexual multiplication within the snail, cercariae are liberated, which penetrate human skin and develop into migrating schistosomulae in order to settle within the mesenteric veins or the perivesical venous plexus of the bladder. Females can produce up to 3000 eggs per day that must passage through the gut or bladder wall in order to be transmitted [78]. The severe morbidity that is associated with schistosomiasis is a result of granulomatous reactions to the highly immunogenic eggs that are backwashed, becoming entrapped in the liver. In a minor proportion of infected individuals, these lesions fail to resolve and may progress to hepatosplenomegaly, chronic hepatic fibrosis, portal hypertension, and ultimately, death [79, 80]. 

### Schistosoma: Migratory Stages

The capacity of eosinophils to adhere to *Schistosoma* spp. was demonstrated decades ago to be dependent on the activation of complement [81]. The *in vitro* killing of schistosomulae required the cytotoxic activity of activated human eosinophils, but not neutrophils, in combination with heat-inactivated sera from infected patients, a process dependent on IgG1 and IgG3 [82-84]. Also, *in vitro *incubation of schistosomulae with eosinophils and specific antibodies prior to their injection into rodents led to a marked reduction of parasite viability [85], demonstrating the importance of eosinophils in direct parasite killing. Release of toxic molecules, such as elastase and hydrogen peroxide is an important mechanism that efficiently kills the parasites, as has been demonstrated for *Fasciola hepatica* [86] and schistosomes, with both molecules being liberated by neutrophils, eosinophils and macrophages [87]. 

Additional involvement of granulocytes in the priming of immune responses has been investigated over the past decades. It is well known that the infection with *Schistosoma* spp. begins with a marked T_H_1 response, shifting towards a strongly T_H_2 polarized response. The first three hours of transformation from cercariae into skin-stage schistosomulae coincides with the release of pre-formed cercarial gland material into the dermis. This heterologous mixture of highly glycosylated secretions contains several proteases to aid dermal penetration but is also implicated in immunomodulation (reviewed in [88]). In primary experimental mouse infections, neutrophils are rapidly recruited to the skin infection site [89, 90], followed by macrophages, Langerhans cells and DC. Fluorescent labeling of gland contents reveals that all these populations phagocytose the released products and subsequently migrate to the skin-draining lymph nodes, delivering and presenting schistosomal antigen to CD4^+^ T cells [90, 91] to initiate a predominantly T_H_1-biased cytokine responses (*i.e*., IFNα , IL-2). Interestingly however, DC exposed to released products prime a T_H_2 response *in vitro* and *in vivo *[92]*. *Indeed, after the onset of patent infection and egg deposition in hepatic and intestinal tissues (see below) the initial prevailing T_H_1 response is modulated in favour of a strongly polarized T_H_2 response (IL-4, IL-5, IL-13) that coincides with peak granuloma formation when the eggs are produced [94] (reviewed by [95, 96]). Excretory/secretory (E/S) products of invading larvae and those secreted by lung-stage schistosomulae, may also contribute to this second phase induction *via* a direct T_H_2 priming effect on DC [92, 97]. Consistent with this, dermal conditioning of antigen-presenting cells by eosinophils during multiple larval infections of the skin further down-regulates the T_H_1 response in a mechanism requiring a recruitment of large numbers of RELM-α + eosinophils providing an enriched T_H_2 cytokine (IL-4, IL-13) skin microenvironment that conditions DC and macrophage populations that can subsequently impact negatively on egg granuloma size in the liver [93].

In non-permissive rats (schistosomes are spontaneously eliminated in the third week of infection), parasite elimination coincides with eosinophilia and elevated IgE [98] and ADCC plays a major role [99]. Because mice and humans are permissive to primary and re-infections, this may potentially reflect the fact that mice, rats and humans show profound divergence in the propensity of eosinophils to degranulate [100, 101] and display differences in eosinophil high affinity IgE receptor expression [102-104]. Vaccination of mice with radio-attenuated, but not normal cercariae, leads to a high level (60-70%) of immunoprotection to subsequent challenge (reviewed by [105]). The site of arrestment of attenuated schistosomulae is in the lungs, but migration from the skin to the lungs is also substantially retarded, pointing towards the priming of protective immunity at one or both sites [106, 107]. The location of challenge attrition, however, is exclusively at the lung migration site (reviewed in [108]). IFNγ-producing CD4^+^ T cells are recruited to this site during challenge infection potentially mediating parasite killing *via* classical activation of macrophages producing iNOS *via* an IFNγ and TNFRI-dependent pathway [109, 110]. However, T_H_2 responses also appear important as IL-4Rα^-/-^ mice are not protected despite strong T_H_1 responses and cellular infiltration into the lungs [111]. Indeed, in T_H_1 deficient mice (IFNγR^-/-^, IL-12p40^-/-^), a significant level of protection (as much as 40%) is evident and coincides with a marked eosinophil influx and elevation in circulating IgE [105]. The direct role of eosinophil ADCC as a mechanism of parasite attrition remains contentious however, as IL-4 or IL-5 depletion of eosinophils and IgE does not reduce vaccination-mediated challenge protection in mice [112].

### *Schistosoma*: Granuloma Formation

Granuloma formation is initiated by release of eggs that are deposited in the liver, intestinal or bladder tissues*.* *S. mansoni* eggs secrete a limited range of molecules [113], which can polarize toward a marked CD4^+^ T cell programmed inflammation with IL-4 and IL-13 [114]; however, CCL3 [115], an activator of granulocytes, is also produced. In *S. mansoni, *the two most abundant egg-secreted products both direct the T_H_2 anti-egg response, but utilize distinct mechanisms. IPSE has been shown to induce basophil release of IL-4 *in vivo*, [72] providing an early innate source of this polarizing T_H_2 cytokine at the infection site, and possibly within the draining mesenteric lymph nodes, as basophils can migrate to secondary lymphoid tissue upon activation. A second molecule, Omega-1, mediates T_H_2 polarization *via* internalization by DC [116, 117]. The T_H_2 priming ability of DC exposed to Omega-1 is dependent on the molecule’s ribonuclease activity, and DC that have internalized Omega-1 display altered cytoskeletal architecture, possibly modifying their antigen presentation efficiency [117]. Because these molecules are restricted to *S. mansoni* [118], distinct T_H_2 polarizing, but as yet undefined processes, must occur in other schistosome species. 

The balance between T_H_1 and T_H_2 cytokines determines the extent of granulomatous inflammation and thus larger granulomas are usually associated with strong anti-egg T_H_2 responses, whereas minimal lesions are observed when T_H_1 cytokines dominate [119]. In humans, variation in colonic granuloma size is positively associated with peripheral blood mononuclear cell responsiveness to egg antigen. T_H_2 responses are critical in the survival of murine hosts during acute-stage egg deposition because the absence of IL-4 signaling is lethal shortly following the onset of egg production [45, 120-122].

In the *S. mansoni *mouse model, egg granuloma formation consists of a dynamic acute phase followed by a chronic phase. The acute phase occurs when anti-egg T_H_2 reactivity is at a maximum both at the deposition sites and within the mesenteric lymph node and spleen (reviewed in [95, 96]). This phase also coincides with maximal granuloma size in hepatic tissue, and peaks at approximately 8 weeks post-infection (3 weeks after the onset of egg deposition). At this stage, within hepatic tissues, eosinophils constitute the majority of cells (>50%) [123] and tend to be most abundant proximal to the egg surface, reflective of their rapid recruitment. The production and release of reactive oxygen species (ROS) probably has detrimental effects on the parasite and the administration of melatonin, a well-known antioxidant, prevented most of the damage that normally occurs in mice infected with *S. mansoni*, *e.g.,* eosinophil infiltration [124]. In contrast, neutrophils comprise a very minor population (between <1-2% in the liver and undetectable in the gut) [123, 125] of the cellular infiltrate in egg granulomas. However, it must be noted that in intestinal granulomas, macrophages are the predominant cell type and a significant, yet reduced, proportion of eosinophils (44% in the colon and 21% in the ileum) are recruited [125]. Despite their preeminence within schistosome egg granulomas, the protective function of eosinophils remains elusive. Whereas in the IL-5 knock out system hepatic granulomas are significantly smaller and IL-13-dependent tissue fibrosis reduced (although the authors examined a later time point, at +16 weeks) [123], eosinophil-lineage-negative mice (ΔdblGATA, TgPHIL) show no impaired survival after the onset of egg production up to 11 weeks post-infection. Strikingly, no difference in granuloma size was observed, demonstrating a compensatory recruitment of other leukocyte populations into granulomas in the absence of eosinophils [23]. In addition, gross hepatic damage and fibrosis were not significantly different in these mice. 

### Cestodes

The Cestoda (cestodes or tapeworms), in common with the Trematoda discussed above, is a class of parasites in the phylum Platyhelminthes. However, unlike the trematodes, the cestodes are universally hermaphroditic. The anatomy of adult cestodes in the major subclass, the Eucestoda, is simple: the rounded scolex features suckers and/or hooks that enable the tapeworm to attach to the wall of the intestine, while the remainder of the body forms the flattened strobila (attaining several metres in length in some species), which is composed of segments or ‘proglottids’. Mature proglottids either release eggs into the gastrointestinal tract, or detach from the strobila and emerge intact from the anus before the eggs are liberated. Once the larvated eggs (oncospheres) or a motile embryonic form are ingested by a suitable intermediate host, the larval metacestode migrates from the intestine into other tissues, where it forms either a spherical or multilobular cyst, or an elongated sparganum. In most species, the lifecycle is completed when this tissue stage is consumed by the definitive host, although some species of tapeworms utilise two intermediate hosts, whereas others have lost the requirement for an intermediate host [78].

In general, adult tapeworms cause little pathology in humans, but the cystic stage can induce life-threatening complications. The species of greatest medical importance are the pig tapeworm (*Taenia solium*), which causes neurocysticercosis, and two species in the genus *Echinococcus* that cause either alveolar (*E. multilocularis*) or cystic (*E. granulosus*) echinococcosis (the latter is also known as ‘hydatid disease’) [78].

Few studies have investigated the role of neutrophils in cestode infections and these cells are rarely observed in association with metacestodes in tissue [126], although they are well represented in infiltrates around intracranial infections with *Mesocestoides corti *in mice [127]. However, neutrophil chemokinesis in response to extracts from *T. taeniaeformis *metacestodes [128] and the killing of cestodes by reactive oxygen species released from neutrophils have been demonstrated *in vitro* [129]. Moreover, protective immunity stimulated by CpG oligodeoxynucleotides in the murine *T. crassiceps* model was associated with neutrophil influx into the site of infection [130]. With respect to basophils, human infections with both *E. granulosus* and *E. multilocularis* induce specific IgE production in which these antibodies often remain bound to the surface of basophils; in this state, they are responsive to a factor released by mononuclear cells that triggers histamine release [131]. Moreover, basophils secrete both IL-4 and IL-13 when stimulated with extracts from *E. multilocularis*, and this response is dependent on surface-bound IgE [132]. Thus, specific histamine secretion and degranulation of basophils stimulated with *Echinococcus *antigens *in vitro* has been developed as a highly sensitive diagnostic test for infection with these parasites [133]. 

Metacestodes are particularly potent inducers of eosinophilia. Early studies in rodent models, using species such as *M. corti, T. taeniaeformis*, *T. crassiceps*, *Hymenolepis nana* and *H. diminuta, *established peripheral eosinophil levels that enabled pioneering functional studies on these cells before the advent of IL-5-overexpressing mice [134]. This eosinophilia usually extended into tissue infiltration around the developing cyst [135]. Secondary challenge of rodents resulted in a rapid eosinophilia that was associated with “eosinophilotactic factors”; these also induced eosinophilia when transferred passively into naive hosts *via* serum [136]. Moreover, cestodes were found to secrete products that had marked chemotactic activity for eosinophils by direct stimulation [137]. Not only recruitment but also the activity of the eosinophil may contribute to resistance to reinfection, as they have been shown to release extracellular oxygen radicals in the intestine of mice infected with *H. nana*, which was associated with larval degradation [138]. 

In pigs naturally infected with cysts of *E. granulosus*, heavy parasitosis was associated with a massive infiltration of eosinophils around nascent cysts, which led to death and resorption of the parasite [139]. This may reflect concomitant immunity that increases with host age [140]. Additionally, mouse strains naturally resistant to *T. taeniaeformis *exhibited potent eosinophil responses to metacestodes in the liver, in which degranulation was clearly associated with parasite degeneration [141, 142]; and eosinophils were also implicated in the resistance of ‘rapid responder’ mice to larval stages of *H. nana *[143] and *M. corti *[144]. In the case of adult tapeworms in the intestine, eosinophils were associated with expulsion of the strobila of *H. diminuta* in the rat, and this process was partially dependent on T-cells, as anti-thymocyte serum reduced both eosinophilia and the destrobiliation response [145]. Similar results were obtained when the survival of larval *T. taeniaeformis *was compared between naturally resistant and severe combined immunodeficiency mouse strains [146]. In mice challenged with protoscoleces from hydatid cysts, a murine strain deficient in the complement component C5 showed higher levels of parasite establishment and larger cysts than did wild-type mice; these differences were associated with reduced levels of eosinophil infiltration in the C5-deficient strain [147]. However, eosinophil degranulation onto the surface of *T. solium* cysts in skeletal muscle or the diaphragm of naturally infected pigs did not cause any obvious damage to the metacestode tegument [148]; although in more recent studies, eosinophils were associated with disrupted parasite vesicular membranes in late-stage porcine muscle cysts of *T. solium *[149] and hydatid cysts in cattle [150]. Conversely, in a time-course experiment performed with *T. taeniaeformis* in the rat, granulocytes closely associated with the surface of the metacestode became pyknotic and degenerated, while cyst development was completely unhindered [151]. In addition, studies using antibody neutralisation or gene knockout of IL-5 in mice infected with *M. corti* have failed to identify any role for eosinophils in mediating host protection [152, 153].

The extent of the ability of eosinophils to control cestode infections may thus be dependent on the innate resistance of the host, the development of concomitant immunity, or effective vaccination. In sheep that have become partially immune to *T. hydatigena *through natural exposure, larvae from an experimental challenge become encircled by layers of eosinophils that are in turn surrounded by T- and B-cells as the immune response progresses [154], while in primary infection, no such infiltration is observed [155]. Immunotherapeutic vaccination with native antigen in pigs naturally infected with *T. solium* resulted in a marked ingression of eosinophils around the metacestodes, and these cells destroyed the developing parasites by degranulating on the tegument [156]. Follow-up studies demonstrated that immunisation with native antigen was effective at eliminating cysts whether the pigs acquired infection before or after vaccination, and in both cases eosinophils were instrumental in parasite destruction [157]. Inoculation of *T. solium *metacestode antigen into mice also induced a pronounced eosinophilia with the production of specific IgE [158]. Interestingly, mice challenged with viable oncospheres of several different species of cestodes showed complete eosinophil-mediated cross-resistance to oncospheres of *H. nana* [159].

It is intriguing that despite this preponderance of data from natural infection in domestic animals and in laboratory models, IL-5 and eosinophilia are actually associated with an increased risk of recurrent infection with *E. granulosus* in humans [160]. Moreover, disease severity in human neurocysticercosis is related to eosinophil numbers, among other factors [161], although this is not surprising since the brain is an immunoprivileged site. In contrast, the positive association between TH-2 responses (including the production of IL-5 from PBMC *in vitro*) and disease severity in humans infected with *E. multilocularis* did not extend to circulating levels of eosinophils, which were not elevated in any patient group [162].

Indirect evidence for a protective role for granulocytes in cestode infections can be inferred from the immunomodulatory molecules expressed by these parasites that interfere with granulocyte function. For instance, both *E. granulosus* and *E. multilocularis *produce “antigen B” (AgB), a large polymeric lipoprotein found in cyst fluid [163]. Components of this complex can inhibit neutrophil chemotaxis and the platelet-activating factor-mediated respiratory burst of these cells *in vitro* [164]; moreover, AgB drives a potent, non-protective Th-2 response that is positively associated with progressive disease in *E. granulosus*-infected patients [165]. A tegumental protein from *E. granulosus*, EgTeg, was also found to inhibit neutrophil chemotaxis and induce IL-4 production from the T-cells of infected patients [166]. These data are consistent with the observed loss of the chemotactic response to parasite antigens in neutrophils recovered from the peritoneal cavity of mice infected with *E. multilocularis *[167]. Furthermore, an antigen extract from *T. solium *metacestodes induced DNA fragmentation and inhibition of phagocytosis in neutrophils *in vitro *[129]. Cestode products that can disrupt eosinophil function have also been identified, such as a cysteine protease secreted by metacestodes of *E. multilocularis* that digests host eotaxin, preventing the infiltration of eosinophils into the peritoneal cavity of infected mice [168]. In addition, prior administration of a ribonucleopeptide from the metacestode of *T. solium* abrogated tissue eosinophilia and parasite destruction in mice subcutaneously implanted with metacestodes [169], while an annexin secreted by this parasite was reported to induce eosinophil apoptosis *in vitro *[170]. This latter finding is also supported by recent data revealing high levels of eosinophil apoptosis in the peritoneum of mice infected with *T. crassiceps *[171]. Thus, cestodes may have evolved immune evasion strategies to limit eosinophil degranulation, as it has been demonstrated that recombinant human ECP can damage protoscoleces of *E. granulosus* *in vitro*. Moreover, in the same study, ECP was detected both on the surface of hydatid cyst walls and in cyst fluid from human-derived parasite material [172].

### Nematodes

The phylum Nematoda (roundworms) is characterized by a cylindrical body shape and includes a number of parasites that are pathogenic in humans and/or livestock. Nematodes are equipped with a cuticle, which requires constant molting during development and this is produced by the hypodermis, a thin lining beneath the cuticle that forms two longitudinal cords medial to a dorsal and ventral cord. They are equipped with longitudinal muscle cells, but unlike the Annelida, they lack circular muscles. A remarkable feature of these parasites is their adaptation to very different tissues of the host, ranging from the gut to the dermal tissues, and from the lymphatics to the coelomic cavities. Therefore, they have developed a variety of migration pathways that can be very simple, as for the order Oxyurida (which undertake out their lifecycle entirely within the gastrointestinal system); or more complex, as for *Ascaris lumbricoides* (which is ingested as an embryonated egg, hatches as an infective larva in the gastrointestinal system, travels through the blood to the lungs, then migrates back into the gastrointestinal system). In contrast with oral uptake, larvae of nematodes such as *Strongyloides stercoralis* or *Necator americanus* enter the host *via* penetration of the skin. Thus the host has had to evolve a range of strategies to combat these diverse parasites [173]. 

### Soil-Transmitted Nematode Infections

Species in the genera *Ascaris* (giant roundworm), *Ancylostoma* and *Necator* (hookworms), *Strongyloides* (threadworms), and *Trichuris* (whipworms) are soil-transmitted parasites that together constitute the most prevalent helminth infections of humans. Among these, *Strongyloides* spp. is the only genus that is able to replicate within the host. Although generally not fatal, high infection rates are associated with chronic morbidity that may include anemia and malnutrition [174]. As for many other helminths, expulsion of gastrointestinal (GI) nematodes is highly dependent on T_H_2 responses and depletion of CD4^+^ T cells leads to delayed expulsion of the parasites (extensively reviewed in [175]). 

Much of our current knowledge derives from model systems, of which *Heligmosomoides polygyrus* in mice has a strictly enteric life cycle, invading the muscularis of the jejunum [176] for a short period, after which it migrates back into the lumen. Infection induces a pronounced infiltration of eosinophils and neutrophils, but also mast cells into the mucosa and submucosa [177]. Evidence for the ability of granulocytes to attack *H.* *polygyrus* (previously known as *Nematospiroides dubius*) larvae was provided by *in vitro* experiments, in which eosinophils and neutrophils from infected animals were able to reduce the infectivity of larvae in the presence of fresh sera [178], suggesting a complement mediated adherence of granulocytes. *In vivo*, neutrophils accumulate immediately around the parasite, followed by alternatively activated macrophages, DC, CD4^+^ T cells and eosinophils [179]. However, in secondary but not primary infections, initiated after anti-helminthic treatment of mice, anti-IL-4 or IL-4Rα treatment blocked polyclonal IgE responses and abrogated protective immunity, despite a substantial increase in the number of eosinophils. In contrast, anti-IL-5 treatment prevented *H. polygyrus*-induced eosinophilia, whereas it did not alter the parasite loads [180]. Therefore, even though granulocytes are associated with memory responses, their importance for parasite expulsion may be minor in this system. This could be due to the fact that once adult, *H. polygyrus* resides within the lumen and other type-2 dependent mechanisms are more important in controlling worm burden, such as mucus production [181]. Similarly, in *T. muris* infection (in which larvae migrate to the cecum and proximal colon and reside in tunnels of actin-rich, brush-border epithelium [182]), ablation of IL-5 with blockade of eosinophil accumulation in a resistant mouse strain did not facilitate a patent infection [183]. Furthermore, mice devoid of Fc-γ receptor were able to expel the parasite, arguing against a role for ADCC in *Trichuris* infection. The same research group recently showed that IL-4-producing eosinophils accumulated in the mesenterial draining lymph nodes, but again, eosinophil-deficient mice efficiently generated CD4^+^-T cells and T_H_2 cytokines resulting in worm expulsion [184]. Data regarding inflammatory responses to *Trichuris* in humans are rare. However, in patients infected with *T. trichiura,* biopsies of colon tissue revealed an infiltration of eosinophils and neutrophils even in light infections and changes in other cell types (other than a reduction in plasma cells) were not observed [185].

*Nippostrongylus brasiliensis* is another parasite residing in the GI tract, but in contrast to the abovementioned nematodes, the lifecycle includes host entry *via* the skin and passage through the lung tissue prior to settlement in the gut. It has been demonstrated that during infection with this species, eosinophilia is due to prolonged survival within tissues, rather than an increased turnover [186]. In this infection, the skin is an important site for immunity against the invading organism. It has been shown *in vitro* that the relative importance of the three pathways (classical, alternative and lectin) of complement activation differs depending on the species of helminth and the host [187-189]. However, when complement-deficiency of all three pathways was produced on an IL-5 transgenic background in order to facilitate the strong eosinophil-dependent resistance to *N. brasiliensis in vivo *[190], this resulted in a reduction in eosinophil accumulation and extracellular EPO activity, but only a minor change in larval numbers recovered from the lung. This indicates the importance of other, complement-independent mechanisms. Furthermore, in mice devoid of eosinophilopoesis (ΔdblGATA mice), resistance to infection with *N. brasiliensis* is abrogated [191]. A more recent study by this group gives weight to the importance of eosinophils explicitly in the skin, as eotaxin- and STAT6-deficient mice on an IL-5 transgenic background showed reduced eosinophil infiltration into the skin and had impaired resistance to *N. brasiliensis,* but not *H. polygyrus, *which has no skin phase in its lifecycle [192]. This suggests that migration *via* the skin is controlled to a much greater degree by granulocyte-mediated defence mechanisms than is colonization of the gut. 

The importance of T_H_2 immunity in infection with *N. brasiliensis* is indubitable, and T cells play a major role in the development of a T_H_2-type environment [193]. The cooperation of lineage marker-negative cells in T_H_2 immunity has been observed in infection with *N. brasiliensis*. Using IL-4- and IL-13-reporter mice to track these cells, a dominant IL-13-producing, innate cell population (nuocytes) expanded in response to the infection, similar to that seen after injection of IL-25 or IL-33. More importantly, inducing this population by administration of IL-25 was sufficient for worm clearance and restored eosinophilia [194]. 

In humans, gut-dwelling helminthes, such as *Ascaris*, *Ancylostoma*, and *Necator* may cause of pulmonary eosinophilia (Löffler´s syndrome) during migration through lung tissue. This disease is characterized by pulmonary infiltrates and peripheral blood eosinophilia and the symptoms are usually mild, but can become severe, depending on the number of ingested eggs [195]. Sputum examinations show eosinophils and Charcot-Leyden crystals [196], and larvae covered with eosinophils have been observed, which may reflect immobilization and subsequent destruction of the parasite [197]. The intensity and selectivity of inflammatory recruitment suggests that granulocytes are not simply responding to tissue injury caused by migrating larvae as a repair mechanism, but are actively targeting or being targeted by the parasites, since parasite-derived chemotactic factors have been reported to recruit eosinophils and neutrophils [198]. Neutrophils have been shown to react quickly in response to nematode body fluids by chemotaxis, activation and superoxide anion production, mediated *via* CXCR1 and CXCR2 (receptors for the neutrophil chemotactic factor IL-8) [199]. Although the role of neutrophils in helminth infections has been understudied compared with that of eosinophils, neutrophils have the ability to release eosinophil chemotactic factors in addition to superoxide anions [5], and thus may contribute to eosinophil recruitment, as has been shown for infection of guinea-pigs with *Schistosoma japonicum* [200].

In infection with *Strongyloides *spp., the role of neutrophils has been evaluated in greater detail. The life cycle of this nematode includes a free-living stage, and infective larvae enter the host by penetration through the skin or gut mucosa, followed by migration through the lungs, where they ascend the tracheobronchial tree to be swallowed before colonizing the small bowel. Thus, a clinical manifestation of such infection in humans is the Löffler syndrome. In immunocompromised hosts, *e.g.,* AIDS patients, strongyloidiasis can lead to hyperinfection syndrome, as protective mechanisms are highly dependent on T cells [195]. Abraham and colleges have investigated the importance of granulocytes in murine infection with *S. stercoralis*. In this infection, eosinophils and/or IL-5 are essential components of the innate protective immune response [201]. In CXCR2-deficient mice that have a severe defect in the recruitment of neutrophils, impaired protection against primary infection with *S. stercoralis* occurred that was comparable to that seen in eosinophil-depleted mice (anti-CCR3 treatment). Interestingly, only neutrophils, but not eosinophils, seemed to be required for the establishment of adaptive immune responses to larvae after immunization [202]. On a molecular level, MBP has been identified to be involved in eosinophil-mediated larval killing and myeloperoxidase in neutrophil-mediated killing [203]. 

### Food-Borne Nematode Infections:* Trichinella*

Among helminth species, *Trichinella* has an important impact on public health and the economy and regulatory controls imposed on all susceptible animal species intended for human consumption have been implemented. Thus, trichinellosis is a major zoonotic disease, which is endemic in less developed countries of Eastern Europe, Asia and Latin America and the global prevalence is estimated in the millions [204], but it occurs also sporadically in developed countries in Europe and in North America, where raw or undercooked pork and wild game may be consumed as delicacies.

*Trichinella* is an intracellular nematode infecting mainly pigs, small rodents and more broadly all mono-gastric mammals. Digestion of meat of infected animals leads to the release of muscle stage 1 larvae that penetrate inside the intestinal epithelial cells of the small intestine where they moult until becoming adults (males and females). There, they mate and females release newborn stage 1 larvae. These invasive larvae migrate from the intestinal epithelium to the skeletal striated muscle cells, their definitive niche in the host (nurse cells) [205]. In response to infection, the host develops a non-specific immune response in the intestinal mucosa followed by a protective response, which will not prevent the parasite from settling in the muscle cell but will block the migration of newborn larvae in case of reinfection by the same species of *Trichinella*. During enteric infection, developing worms damage columnar epithelium, depositing their shed cuticula. *In vitro* systems have allowed analysis of the response of epithelial cells to infection, however in the absence of other host cells and tissues. Despite the destruction of epithelial cells, co-culture of muscle larvae induced up-regulation of transcripts for the proinflammatory mediators IL-1β and chemokines IL-8 and ENA-78 [206]. Infections in humans have been associated with eosinophils and T_H_2-type responses [205] and re-stimulation of peripheral blood mononuclear cells showed strong IL-5 responses at the mRNA and protein level. Importantly, a strong correlation exists between levels of IgE and blood eosinophilia during the first period of infection. A massive reduction of eosinophils occurs during the acute stage of trichinellosis and this has been shown to predict the severity of the outcome of infection. This phenomenon may be due to a massive peripheral migration [207] and gives weight to the importance of eosinophils in pathology. There is also evidence that eosinophils can kill *T. spiralis* larvae *in vitro* [208]. However, a role for eosinophils in host defense against *T. spiralis* *in vivo* is not convincing as the evidence is contradictory. Although infection of rodents with *T. spiralis* stimulated a basophilia as well as an eosinophilia [209], the parasite survival is not or marginally modified during a primary infection in IL-5-deficient mice, IL-5 transgenic mice, or in mice depleted of eosinophils with specific antibodies [210]. In addition, CCR3-deficient mice do not recruit eosinophils to nurse cells, and the number of necrotic nurse cells on histologic sections of tongue decreased [211]. Although intestinal infection with *T. spiralis* is not altered by eosinophil deficiency, eosinophil deficiency limits parasite destruction in muscle [212]. In this study, two models of eosinophil depletion (Δdbl-GATA and TgPHIL mice) were used to study muscle inflammation during *T. spiralis* infection. Eosinophils are prominent in infiltrates surrounding infected muscle cells of wild-type mice whereas, in both models infiltrates completely lack eosinophils. These granulocytes are also absent in the blood of infected mice, in contrast to wild-type mice. The recovery rate of *T. spiralis* muscle larvae was lower in these both strains of mice, by 60-70% in TgPHIL mice and 48% in Δdbl-GATA mice respectively. In addition, the T_H_1 response was enhanced (increased levels of IFNγ in *in vitro* lymph node cell culture) and the T_H_2 response downregulated (decreased IL-4 in *in vitro* culture). Larval survival improved when mice were treated with inhibitors of inducible NO synthase, implicating the NO pathway in *T. spiralis* clearance. These results show that muscle larvae are damaged by an immune response driven by T_H_1 cells, which seem to be downregulated by eosinophils. Thus, eosinophils may play a dual role in *T. spiralis* infection, with both effector and regulatory function. 

### Humans as Non-Permissive Hosts

In several other cases of food-borne nematode infections, the parasite is unable to complete its lifecycle in humans. In unnatural hosts, defence mechanisms are usually stronger due to the fact that helminth-induced downregulation of the immune system is part of the evolutionary co-adaptation process. As a corollary to this immune dysregulation, serious complications can result from immune reactions to the migrating larvae. *Angiostrongylus cantonensis*, a species widespread in South-East Asia and the Pacific, naturally parasitizes the pulmonary artery of rats. First-stage larvae in rat faeces infect the molluscan intermediate host (various species of slugs and snails), and humans can become infected by ingesting raw or undercooked molluscs, paratenic hosts (commonly prawns or crabs), or unwashed vegetables directly contaminated with infective larvae. In rats, the larvae migrate to the brain before establishing patent infections in the pulmonary arteries; however, in humans, infection terminates with the immature adult stage in the central nervous system. Angiostrongyliasis is the leading cause of human eosinophilic meningitis, and animal studies have demonstrated that the pathogenesis of the disease is clearly linked to the non-permissive nature of the human host. For instance, EPO levels in cerebrospinal fluid (CSF) were significantly higher in infected guinea-pigs (non-permissive) compared with rat (permissive) hosts [213]. Moreover, when young adult worms were transferred into the pulmonary arteries of non-permissive hosts, degenerative changes associated with eosinophil infiltration and the deposition of EPO onto the worm cuticle were observed, whereas no such cellular reaction was provoked in rats [214]. In the cerebellum, eosinophil degranulation in non-permissive hosts was apparently responsible for ‘bystander’ damage to host Purkinje cells [214].

More recent studies have demonstrated that the pathophysiology in non-permissive hosts is not only associated with eosinophil degranulation *per se*, but also with the levels of plasminogen activators and matrix metalloproteinase-9 (MMP-9) [215]. This enzyme has been associated with eosinophils in the mouse model [216] and was specifically localized to the ‘small’ granules [217]. An imbalance between MMP-9 and tissue inhibitors of metalloproteinases in the subarachnoid space of infected mice may be the key host reaction that leads to manifestations of the disease [218]. Analysis of cytokine responses in the CSF of mice detected IL-4 and IL-5 by two to three weeks post-infection, which were expressed by T-cells but not eosinophils [219]; while eotaxin and macrophage inflammatory protein-1 in murine CSF were chemotactic for eosinophils *in vitro *[220]. In humans, the concentrations of eotaxin-2 [221], IL-5, IL-10 and TNF-α are correlated with eosinophilia [222]. Accordingly, treatment of infected mice with an anti-CCR3 monoclonal antibody decreased eosinophil infiltration into the CSF, and the levels of eotaxin and IL-5, as well as disease severity, were significantly reduced [223]. However, somatic extracts from young adult worms contain at least two proteins that have direct chemotactic activity for guinea pig eosinophils [224].

Early studies using eosinophils *in vitro *suggested that immunological protection against *A. cantonensis* was mediated by ADCC. With the advent of the genetic knockout era, the critical role of IL-5 and eosinophilia in controlling worm burden in mice was confirmed. Thus, angiostrongyliasis is a classic case of a helminthic infection in which eosinophils are both the key effector of protective immunity and the primary cause of disease symptoms in non-permissive hosts. Over evolutionary history, the natural host appears to have reached equilibrium with the parasite, since eosinophilia in the CSF could be induced in cerebrally-infected rats by injection of antigens from first-stage larvae or eggs, whereas extracts from young adult worms (*i.e.*, the stage that affects the brain) failed to stimulate this response [225]. Indeed, the severity of disease in mice can be ameliorated by the administration of IL-12, which was shown to successfully reduce meningeal eosinophilia *via* a switch to a type-1 response, in combination with an anthelminthic (mebendazole). Importantly, IL-12 alone was less effective, as the viable worms were still able to cause mechanical damage to the parenchyma [226]. The efficacy of this combination treatment may result, in part, from its ability to reverse the observed resistance of murine eosinophils to apoptosis in the CSF [227].

Another food-borne nematode infection, although with a more global distribution, is anisakiasis, caused by members of the genus *Anisakis*. The natural definitive hosts of *Anisakis *spp. are marine mammals, but humans can become transiently infected by consumption of raw or undercooked fish (paratenic hosts) containing third-stage larvae. Thus, in common with angiostrongyliasis, humans are non-permissive hosts, although development of the nematode is normally truncated at an earlier stage (*i.e., *the L4) within the gastrointestinal mucosa. Infection is associated with gastrointestinal symptoms and allergic-type immunological reactions [228]. Crude extracts from *Anisakis* larvae exhibited potent chemotactic activity for eosinophils *in vitro* and *in vivo* [229], and induced an eosinophilic phlegmonous reaction in the ileum of rabbits without prior exposure [230]. However, live L3 implanted into the abdominal cavity of mice generated an intense infiltration of neutrophils, which was not displaced by eosinophils until granulomas matured from 14 days post-infection [231]. Further studies on the granulomas revealed that although eosinophils degranulated onto the cuticle of the parasite, they lacked the ability to kill the larvae; whereas macrophage adherence was associated with gross damage to the parasite surface [232]. Immunopathogenesis in humans was associated with transcript levels for MBP, eotaxin and inducible nitric oxide synthase (iNOS) in eosinophilic infiltrates within the intestinal wall; while ECP and EDN concentrations in the sera of patients were also elevated [233]. Importantly, allergic responses to *Anisakis* spp. are not dependent on exposure to live larvae, and thus sensitisation to parasite antigens can occur following consumption of fish containing dead parasites, or even *via* inhalation or skin contact with allergens in fish-processing plants [228, 234]. In a mouse model of allergic inflammation, intranasal administration of E/S material from *Anisakis *L3 triggered the production of IL-17, IL-6 and the neutrophilic chemokine CXCL1, in conjunction with neutrophil infiltration into the lung. Furthermore, the induction of IL-6 and CXCL1 in mouse embryonic fibroblast cells by *Anisakis *ES products was dependent on TLR3 (Table **1**), although surprisingly, RNase treatment of the E/S did not abrogate this effect [235]. The key *Anisakis *allergen (Ani s 4) has been identified as a cystatin, and the recombinant protein was capable of activating basophils from allergic patients [236]. Indeed, basophil activation assays have been employed both to diagnose *Anisakis* allergy and to detect allergens in fish muscle [237].

### Beneficial and Detrimental Effects of Granulocytes in Helminth Infections: Lessons from Filariasis

Among the helminths, filarial nematodes constitute the greatest burden of human morbidity worldwide. These parasites are transmitted by haematophagous arthropod vectors, such as mosquitoes and blackflies (*Simulium* spp.), which become infected by ingesting first-stage larvae (microfilariae) from the blood or skin of the definitive human host. Within the intermediate host, the larvae moult twice and become infective third-stage larvae (L3) that migrate to the head of the vector. The L3 are deposited on the skin of the new definitive host during a blood meal, and migrate *via* the lymphatics to their specific tissue predilection site, where they moult twice prior to maturation as dioecious adults. Fertilised female worms are ovoviviparous, giving birth to fully formed microfilariae rather than eggs. Filarial worms are responsible for three major neglected tropical diseases: lymphatic filariasis (caused by *Wuchereria bancrofti* and *Brugia *spp.), in which the adult worms reside in the lymphatic vessels; onchocerciasis (caused by *O. volvulus*), in which the adult worms are found in subcutaneous nodules; and loiasis (caused by *Loa loa*), where adult worms migrate throughout the connective tissues. In lymphatic filariasis, it is the adult worms that are responsible for the symptoms of the disease (lymphoedema and hydrocoele); while in onchocerciasis, the nodules are benign and it is the death of microfilariae in the skin and eyes that triggers severe dermatitis and visual impairment (River Blindness). In loiasis, the migrating adult worms can cause relatively minor pathology (Calabar swellings).

Several rodent models of filariasis have been developed, including those using the human parasites *B. malayi* and *O. volvulus*, which undergo only partial development in laboratory mice; alongside the rodent parasites *Acanthocheilonema viteae* (a natural parasite of the jird, *Meriones unguiculatus*) and *Litomosoides sigmodontis* (a natural parasite of the cotton rat, *Sigmodon hispidus*). The latter is a particularly powerful model species, as it can complete its development in BALB/c mice, exposing the complete filarial lifecycle to the tractable technologies of murine immunology [238]. Several studies using transgenic mice have sought to determine the role of granulocytes in protective immunity against filarial nematodes. For instance, in mice lacking B-cell maturation and antibody production (µMT strain), it has been demonstrated that the vast majority of incoming L3 are killed by an innate immune response dominated by neutrophils [239]. However, a clear consensus has emerged from experiments in several of the above rodent models that the protective immunity induced by irradiated L3 is mediated by eosinophils and antibody within two days of challenge [240-242]. Hence, the potency of this vaccination method was abated both in µMT mice, in which eosinophils were recruited to the site of infection but failed to degranulate [239], and in mice depleted of eosinophils or IgE [243]. In contrast, in primary infections in mice over-expressing IL-5, targeting of the larval stages by eosinophils occurs from day 10 post-infection, and the young adults become embedded in eosinophil-rich granulomas. The difference in the timing of killing relative to irradiated vaccination can be attributed to the delay required to produce specific antibodies in a primary infection [244]. The precise effector mechanism deployed by eosinophils when targeting filariae has not been fully elucidated, although knockout of EPO or MBP led to increased worm burdens of *L. sigmodontis *in mice, suggesting a role for these granule proteins in defense against the worms. It is not known whether this was primarily due to reduced effector capacity of degranulating eosinophils, or an indirect mechanism involving altered levels of IL-10, IL-5 and/or IL-4, which were affected by knockout of the granule proteins [245].

Several lines of evidence from the *L. sigmodontis *model indicate that neutrophils also have an important role in control of the adult worm burden. Thus, when the accumulation of neutrophils in granulomas around the adult worms was abrogated by neutralization of G-CSF, parasite killing was inhibited, despite the normal presence of eosinophils in the granulomas [246]. Knockout of IFN-γ greatly reduced the effector function of neutrophils and resulted in an increased worm burden, probably because these mice also exhibited decreased production of the key neutrophil activator, TNF-α [247]. When combined with an additional knockout of IL-5, neutrophil chemotaxis and phagocytosis (assessed *in vitro*) were even more impaired, worm burdens were higher than in single knockout mice, and TNF-α production was markedly reduced [248]. In contrast, only a single study has attempted to determine the role of basophils in protective immunity against filarial infection. Depletion of these cells had no effect on adult worm numbers in the *L. sigmodontis* model, despite a significant reduction in IgE levels, eosinophilia and production of IL-4 in treated mice [76].

Mouse models of filariasis are hampered by the lack of immunopathology generated during infection, and thus analysis of an extremely important aspect of the human diseases is largely restricted to clinical studies. However, keratitis (resembling that observed in onchocerciasis) can be induced in mice by direct injection of filarial extracts into the cornea [249]. Studies using this model have shown that neutrophil accumulation occurs early after injection of *O. volvulus* crude antigen, independently of adaptive immune responses [250]. This can be attributed to a particularity of filarial nematodes, in which many (but not all) species contain endosymbionts of the genus *Wolbachia *[251]. Consequently, depletion of these bacteria from filarial extracts prevented both innate recruitment of neutrophils and ocular pathology in the mouse model. Granulocytes are also involved in secondary immune responses in the eye, which are induced when injection of the corneal stroma is preceded by subcutaneous immunization of the mice with filarial antigen. In this situation, both neutrophils and eosinophils infiltrate the cornea, although P-selectin-dependent eosinophil influx occurs later than the neutrophil response [252]. Ocular damage was abrogated in CXCR2 knockout mice [253], in which neutrophil recruitment was prevented, but not in P-selectin-deficient mice, which lacked eosinophilic infiltrates [252]. In contrast to neutrophil accumulation, eosinophil influx into the corneal stroma is dependent on adaptive responses; *i.e.*, filarial-specific antibodies [254] and a functional T-cell response [255]. 

Human onchocerciasis manifests as a disease spectrum between two clinical “poles”: the generalized or hyporesponsive form (which is more common), and the localized or hyperreactive form (if unilaterally predominant also known as “Sowda”). In the hyporesponsive form, the skin contains millions of microfilariae and more than 50 adult worms may be present in a single individual. In contrast, the Sowda form is characterized by extremely low densities of dermal microfilariae, low numbers of adult worms in enlarged inflammatory nodules, and severe lichenification of the skin. The pathogenesis of Sowda can be attributed to a more potent T_H_2 response than is observed in hyporesponsive onchocerciasis, as revealed by higher levels of parasite-specific IgE [256], elevated numbers of eosinophils and mast cells in nodules [257], increased concentrations of EDN in serum [258], and a significant association with a polymorphism in the IL-13 gene [259], which could lead to enhanced induction of the T_H_2 pathway. Moreover, eosinophils from Sowda patients exhibited greater chemotactic responses to platelet-activating factor (PAF) *in vitro* than did eosinophils from patients with the hyporesponsive form; whereas neutrophil chemotaxis to PAF and the synthetic peptide fMLP was diminished in Sowda compared with the migration observed with neutrophils from hyporesponsive individuals [260]. In lymphatic filariasis, a rare clinical manifestation known as “tropical pulmonary eosinophilia” (TPE) represents another form of extreme T_H_2 polarization induced by hyperreactivity to microfilariae; this syndrome is associated with massive accumulations of eosinophils in the lung, the site where microfilariae are killed as well as peripherally [261]. In addition, IgE antibodies against filarial γ-glutamyl transpeptidase have been implicated in the pathogenesis of TPE, as they react with the host homologue of this molecule in the pulmonary epithelium, leading to autoimmunity [262]. Recently, the processes underlying the clinical responsiveness of TPE to treatment with diethylcarbamazine (DEC) were investigated in a mouse model of airway sensitization, and IL-5-dependent eosinophilopoiesis was found to be suppressed by this drug *via* a mechanism requiring both iNOS and Fas ligand [263]. 

An abundance of literature demonstrates that granulocytes are the key players mediating microfilarial killing following anthelminthic treatment. Interestingly, two of the most important compounds currently used in mass control programs for filarial infections, ivermectin and DEC, do not affect the viability of microfilariae *in vitro* at concentrations that are effective *in vivo*. This is probably because of a combination of subtle, sub-lethal effects on the microfilarial sheath [264] and/or secretory apparatus [265] together with immunopotentiating activity [266, 267]. Interestingly, for ivermectin, a dose dependent effect on the generation of eosinophilic-derived toxic oxygen intermediates was found, *i.e.* when applied in low concentrations, the release of these molecules was enhanced, whereas it was reduced at higher doses of the drug [268]. Among many reactive oxygen species, hydrogen peroxide, which can be produced by granulocytes, was the most effective in killing microfilariae of *O. cervicalis *[269]. The administration of DEC or ivermectin in human patients leads to a rapid (*i.e., *within 1 – 3 days) accumulation of microfilariae in the lymph nodes, to which eosinophils adhere and degranulate, destroying the target [270, 271]. In onchocerciasis, chemotherapy with DEC induces the “Mazzotti reaction”, a syndrome which includes fever, urticaria and hypotension. This adverse reaction is associated with elevated levels of MBP and EDN in serum and the release of MBP by eosinophils in the skin [272]. Moreover, neutrophils and their granule contents are also abundant in the microabscesses surrounding dermal microfilariae following DEC treatment [273]. Since DEC also kills the microfilariae in the eye, leading to an influx of inflammatory cells that mediate keratitis and other eye damage, it is now contraindicated as chemotherapy for onchocerciasis. 

Adverse events following the chemotherapy of filarial infections have also been linked to the rapid release of *Wolbachia *endosymbionts from dying worms. Treatment with DEC led to significantly elevated levels of *Wolbachia *DNA in serum, which were correlated with blood neutrophil counts and the concentration of calprotectin [274]. Furthermore, reactions scores, neutrophil counts and serum calgranulin B levels were positively correlated with *Wolbachia* DNA concentrations after ivermectin chemotherapy. Accordingly, in patients infected with *B. malayi*, severe adverse reactions associated with DEC and albendazole treatment can be mitigated by prior administration of doxycycline [275], which depletes *Wolbachia *from filarial adult worms and microfilariae [276]. Since antibiotic chemotherapy of onchocerciasis kills the adult worms slowly *via* an indirect mechanism (*i.e., *targeting of the endosymbiont [277, 278]), these regimens do not generate adverse reactions triggered by the sudden release of large quantities of filarial and bacterial antigens. Within the collagenous nodules containing the adult worms, the presence of the endobacteria is associated with extensive neutrophil infiltration without tissue damage, profuse pus formation or necrosis; whereas eosinophils are normally scarce [279, 280]. In the bovine model of onchocerciasis, *O. ochengi*, oxytetracycline chemotherapy results in the depletion of *Wolbachia*, a gradual decline in the nodular neutrophil population, and a massive infiltration of eosinophils [280]. These cells were observedby histology to contribute to the killing of the adult worms by degranulating on the cuticle. Importantly, this sequence of events is not seen following administration of an adulticidal drug with a direct mode of action (*i.e.*, an anthelminthic without an antibacterial effect), indicating that the symbiosis between *Wolbachia *and *O. ochengi *may involve protection of the worms from eosinophil effector mechanisms through the recruitment of a less effective neutrophil response [281].

## CONCLUDING REMARKS

Despite the enormous strides made in our knowledge of the biology of granulocytes and their interactions with helminths over the past decades, new data have not allowed the parasitology community to develop a ‘grand synthesis’ that definitively explains this relationship. Indeed, perhaps such a ‘Holy Grail’ was always an illusory goal considering the great diversity of helminths, which bridge two invertebrate phyla with an estimated evolutionary divergence time of more than one billion years [282]. Moreover, humans are afflicted by a considerable variety of helminth species that often undergo extensive morphological changes within the lifecycle, migrating through different tissues to reach their final predilection site. On the host side of the equation, the granulocytes that were once perceived to be terminally differentiated, ‘kamikaze’ effector cells are now recognized to be considerably more sophisticated, in that they can influence the initial development of acquired immune responses through antigen presentation, and can modulate the response of other leukocytes in processes such as wound healing. Once the intrinsic differences between the immune responses of different mammalian hosts are also considered, the wonder is that any consistent patterns have emerged from analysis of granulocyte-helminth interactions.

The neglected basophil has recently experienced its own period in the limelight in the context of helminth infections [7]. While the potential role of basophils as APCs that can induce T_H_2 responses is important and intriguing, they do not appear to have an indispensable function in this respect. Moreover, few studies have established an effector function for these cells against helminths, although whether this simply reflects a relative paucity of data relative to eosinophils remains to be determined. However, it is clear that basophils are responsible for the amplification of T_H_2 responses in helminth infections and are the key player in allergic hypersensitivity reactions to worm antigens.

Consequently, the eosinophil remains essentially unchallenged as the cell at the centrepiece of the granulocyte-helminth relationship. New studies continue to reinforce the paradigm that eosinophils are the key granulocyte mediating protective immunity in the adaptive context in humans and large animals, particularly with regard to species with tissue-migrating stages. Immunity to cestodes in humans may represent an exception to this pattern, but this a field where few clinical studies have been performed, and of the three groups of helminths, our knowledge of the immunology of cestode infections remains the least well developed. Conversely, evidence from mouse models has often failed to demonstrate a convincing role for eosinophils in conferring protection. However, it is important to note a key difference between human and mouse eosinophils: the latter poorly express receptors for IgE (Fc-ε; [283]). Thus, although murine eosinophils can undergo the respiratory burst when Fc-γ receptors are ligated by an opsonised target [283], synergism between these cells and parasite-specific IgE is not a mechanism that is available to the murine host. One explanation for these differences could be that the evolution of effector function between host species has led murine neutrophils to exhibit a role which overlaps with that of human eosinophils, as the former are often associated with adaptive immunity to helminths in mouse models. In contrast, in helminth infections of humans, most studies suggest that neutrophils are only important during the innate response. The problems of interpreting eosinophil responses to helminths in mouse models have been discussed in detail by other researchers [284, 285], and a key factor that may lead to underestimation of the importance of eosinophils in acquired immunity is the potency of innate responses directed against helminths that are not natural parasites of mice. Thus, the evidence from mouse models must always be assessed in relation to the specific host-parasite relationship, and data from natural mouse-helminth systems may be more informative than artificial infection of mice with human parasites that lack a natural rodent reservoir. However, data from non-permissive hosts are necessary to aid interpretation of the human response to incidental infection with worms such as *Anisakis *spp. and *Angiostrongylus*.

The evidence for the singular contribution of eosinophils to immunopathology in helminth infections is unequivocal. Indeed, it is well recognized that the mechanisms which eosinophils deploy to destroy a target can lead to damage of host tissue, unless this process is tightly regulated. The degree of immunopathology that a helminth infection induces in the human host is dependent on two principal factors. Firstly, when humans are non-permissive hosts, the risk of immunopathology is increased (on those rare occasions when the parasite overcomes innate defenses), relative to infection with a parasite capable of normal development. The severity of disease manifestations is then determined by the migration route of the worm and the site of transient establishment. Thus, helminths that lodge in immunoprivileged tissues such as the eye or brain are more likely to invoke granulocyte-mediated damage than worms located in the gut. Secondly, host genetics underlying atopy are critical in determining the balance between protection and immunopathology in helminth infections, as exemplified by Sowda in onchocerciasis and allergic responses to *Anisakis *proteins. Perhaps the most informative example of the fine balance between the beneficial and pathological roles of eosinophils (and other granulocytes) is the chemotherapy of filarial infections, where these cells are activated and effectively clear microfilariae, but simultaneously release products that can drive severe adverse events.

Recent data indicate that the relationship between helminths and eosinophils has evolved to the point where worms ‘perceive’ these cells as a developmental cue to accelerate their progression through the lifecycle in partially immune hosts. Thus, in mice that mount a vigorous eosinophil response during larval entry, surviving adult worms of *L. sigmodontis* exhibit an accelerated growth [244], reach patency earlier, and produce more microfilariae, relative to worm development in hosts that lack such a response [286]. A related observation was made in mice infected with *T. spiralis*, in which larval killing was enhanced in eosinophil-deficient mice [287]. Perhaps these studies represent the most pertinent answers to the question, “Who is calling the shots?” that have been performed to date. 

## Figures and Tables

**Table 1.** Toll-Like Receptors Expressed by Human Granulocytes and their Natural Ligands

TLR	Ligands	Associated pathogens*	Granulocyte expression#
TLR-1	Triacylated lipoproteins	Bacteria	Neutrophils, eosinophils [291]
TLR-2	Lipoproteins	Bacteria#	Neutrophils [292], Basophils [293], Eosinophils [291]
	Zymosan	Fungi	
	Ara-lipoarabinomannan	Mycobacteria	
	Lysophosphatidylcholine (54)	**Schistosomes**	
	Lipotechoic acid	Gram +ve bacteria	
	Chitin (294; 290)	Fungi, protists, ** nematodes**, arthropods	
TLR-3	dsRNA	Viruses	Eosinophils [295]
TLR-4	Lipopolysaccharide	Gram -ve bacteria	Neutrophils [291], Basophils [293], Eosinophils [296]
	Heat-shock proteins	Various	
	Lacto-*N*-fucopentaose III (55)	**Schistosomes**	
	Glycoprotein ES-62 (58)	**Filarial nematodes**	
TLR-5	Flagellin	Bacteria	Neutrophils [291], Eosinophils [296]
TLR-6	Diacylated lipoproteins	Mollicutes and rickettsiae#	Neutrophils [297], Eosinophils [296]
TLR-7	ssRNA	Viruses	Eosinophils [298], Neutrophils [291]
TLR-8	ssRNA	Viruses	Neutrophils [299]
TLR-9	Unmethylated CpG oligodeoxynucleotides	Bacteria, viruses	Neutrophils [289], Basophils [293], Eosinophils [291]
TLR-10	Unknown	**-**	Eosinophils [291], Basophils [293], Neutrophils [291]

* Helminth pathogens are indicated in bold type.

# The Wolbachia endobacteria of filarial nematodes activate TLR-2 and TLR-6 [59, 60].

**Table 2.** The Role of Granulocytes in Helminth Infection in Animals and Humans

	Trematodes	Cestodes	Nematodes
**Host protection**			
*In vitro* damage or killing	* Schistosoma* spp. (81-84, 87) *Fasciola* *hepatica* (86)		* Heligmosomoides polygyrus* (178) * Trichinella spirialis* (208)
*In vivo* damage or killing	* Schistosoma mansoni* (85)	* Hymenolepis* spp. (138, 145, 159) *Taenia *spp. (141, 142, 154, 156, 157)	* Heligmosomoides polygyrus* (180) * Litomosoides sigmodontis* (239, 245, 246, 248) * Nippostrongylus brasiliensis* (71, 75, 191,192, 194) *Trichuris muris *(19) * Strongyloides stercoralis* (202) * Onchocerca ochengi* (280, 281) * Onchocerca volvulus* (243)
Antigen presentation	* *		*Brugia malayi *(66, 67) * Strongyloides stercoralis* (68, 69)
Granulocyte activation and induction of T_H_2 responses	* Schistosoma* *mansoni*, IPSE (72-74) * Schistosoma mansoni* (90, 91, 93) * *	* Cysticercus* *cellulosae* (129) * Echinococcus *spp*.* (132)	* Litomosoides sigmodontis* (76) * Nippostrongylus brasiliensis* (53, 75)
Granulocyte recruitment	* Schistosoma* *mansoni*, skin (89, 90, 93, 123, 124)	* Echinococcus* *granulosus* (139) * Mesocestoides corti* (127, 144) *Taenia *spp*.* (128, 130, 135, 136, 137, 149)	*Anisakis simplex* (229-231, 236) * Heligmosomoides polygyrus* (179) * Onchocerca volvulus* (279) *Trichuris trichiura* (185)
Parasite establishment			
Inhibition of granulocyte recruitment and function	* Schistosoma mansoni*, lyso-PS (56)	* Echinococcus multilocularis* (167, 168) *Taenia solium* (129, 169) *Taenia crassiceps* (171)	* Trichinella spiralis* (212) * Acanthocheilonema viteae*, ES-62 (58)
Immunopathology			
Association with pathology	* Schistosoma mansoni* (93, 123, 124)		*Anisakis simplex* (213, 225) * Onchocerca volvulus*/*Wolbachia *(252, 253) * Onchocerca volvulus *(256-258, 272, 288) *Brugia malayi* (262)
